# Event-Related Desynchronization During Mirror Visual Feedback: A Comparison of Older Adults and People After Stroke

**DOI:** 10.3389/fnhum.2021.629592

**Published:** 2021-05-31

**Authors:** Kenneth N. K. Fong, K. H. Ting, Jack J. Q. Zhang, Christina S. F. Yau, Leonard S. W. Li

**Affiliations:** ^1^Department of Rehabilitation Sciences, The Hong Kong Polytechnic University, Kowloon, Hong Kong; ^2^University Research Facility in Behavioral and Systems Neuroscience, The Hong Kong Polytechnic University, Kowloon, Hong Kong; ^3^Tung Wah Hospital, Hospital Authority, Hong Kong, Hong Kong

**Keywords:** mirror visual feedback, occupational therapy, mirror neuron, event-related desynchronization, stroke

## Abstract

Event-related desynchronization (ERD), as a proxy for mirror neuron activity, has been used as a neurophysiological marker for motor execution after mirror visual feedback (MVF). Using EEG, this study investigated ERD upon the immediate effects of single-session MVF in unimanual arm movements compared with the ERD effects occurring without a mirror, in two groups: stroke patients with left hemiplegia and their healthy counterparts. During EEG recordings, each group performed one session of mirror therapy training in three task conditions: with a mirror, with no mirror, and with a covered mirror. An asymmetry index was calculated from the subtraction of the event-related spectrum perturbations between the C3 and C4 electrodes located over the sensorimotor cortices contralateral and ipsilateral to the moved arm. Results of the effect of task versus group in contralateral and ipsilateral motor areas showed that there was a significant effect of task condition at the contralateral motor area in the high beta band (17–35 Hz) at C3. High beta ERD showed that the suppression was greater over the contralateral hemisphere than it was over the ipsilateral hemisphere in both study groups. The magnitude of low beta (12–16 Hz) ERD in patients with stroke was more suppressed in contralesional C3 under the no mirror compared to that of the covered mirror and similarly more suppressed in ipsilesional C4 ERD under the no mirror compared to that of the mirror condition. The correlation analysis revealed that the magnitude of ERSP power correlated significantly with arm severity in the low and high beta bands in patients with stroke, and a higher asymmetry index in the low beta band was associated with higher arm functioning under the no-mirror condition. There was a shift in sensorimotor ERD toward the contralateral hemisphere as induced by MVF accompanying unimanual movement in both stroke patients and healthy controls. The use of ERD in the low beta band as a neurophysiological marker to indicate the relationships between the amount of MVF-induced ERD attenuation and motor severity, and the outcome indicator for improving stroke patients’ neuroplasticity in clinical trials using MVF are warranted to be explored in the future.

## Introduction

Hemiparetic upper limb impairment is a leading cause of long-term physical disability after stroke. Among stroke survivors, 70% experience permanent upper extremity hemiplegia (Jørgense et al., 2000), and 33–60% will continue to have no function at 6 months post-stroke (Kwakkel and Kollen, 2013). Often, arms recover slowly, and partial to full non-use of an arm is common even when stroke survivors can walk independently. Facilitation of motor relearning in order to elicit positive neuroplasticity of the damaged brain area during the rehabilitation of a hemiparetic arm has always been a challenging task for occupational therapy (Stoykov and Madhavan, 2015). Mirror therapy has been used by occupational therapists as an effective and cost-effective intervention for arm hemiparesis following stroke. Evidence shows that it can benefit stroke patients in their arm recovery at the subacute stage (Toh and Fong, 2012). One of the possible explanations is that the parietal–frontal area encompasses the so-called human mirror neuron system (MNS), which can be activated during both the observation and execution of movements (Rizzolatti and Craighero, 2004).

Mirror neurons were first discovered in a monkey experiment when the monkey do or observe an action (Rizzolatti and Craighero, 2004). Event-related desynchronization (ERD) – that is, by power suppression over frequency bands on an electroencephalogram (EEG) – has been indicated traditionally by a decrease in the amplitude of mu rhythms over the sensorimotor cortex, as recorded by electroencephalography. ERD can be interpreted as an electrophysiological correlate of an increased cortical excitability or an activated cortical area (Neuper and Pfurtscheller, 2001). Therefore, this phenomenon has been used as a neurophysiological marker for the activation of mirror neurons, owing to its previously defined physiological properties (Pineda et al., 2000; Muthukumaraswamy et al., 2004; Oberman et al., 2005). Such oscillations are based on neural substrates during the observation and execution of a motor act and are also associated with other human functions, such as imitation, language, and the like (Rizzolatti and Craighero, 2004; Binder et al., 2017; Perry et al., 2017). The core mirror neuron system (MNS) serves as a neural substrate to achieve the transformation of visual information into cortical areas for motor execution (Rizzolatti and Sinigaglia, 2010). The MNS is thought to be found in the frontal and parietal lobes – in the primary motor cortex (M1), the premotor cortex (PMC), the inferior frontal gyrus (IFG), and the inferior parietal lobe (IPL) – and to involve interactions between vision, motor commands, and proprioception for motor learning (Deconinck et al., 2015).

According to our recent review, the MNS is thought to be activated by the optical illusion of movement, such as during mirror visual feedback (MVF) or action observation (AO) (Zhang et al., 2018). A review of the literature on the long-term effects of action observation and MVF in stroke also concluded that with MVF, both action observation and action execution could enhance the magnitude of ERD in the sensorimotor cortex as recorded by an EEG and also could enhance the motor evoked potentials (MEPs) elicited by transcranial magnetic stimulation (TMS) (Kang et al., 2011, 2012; Liepert et al., 2014; Marangon et al., 2014). The ERD over the sensorimotor cortex could also be suppressed during action observation (Frenkel-Toledo et al., 2014, 2016; Perry et al., 2017). Therefore, ERD can serve as a neurophysiological marker of sensorimotor activation or a measure of visuomotor transformation, which could be induced through either a movement execution or a movement observation in occupational therapy treatment such as MVF (Zhang et al., 2018).

Mirror visual feedback also causes instant neuromodulatory effects of increased activation of the ipsilateral superior temporal gyrus (STG) (Matthys et al., 2009) and the PMC (Ramachandran and Altschuler, 2009) during training. The STG and the PMC serve as a network for the imitation of biological motion and the acquisition of motor skills (Deconinck et al., 2015). Previous studies have been conducted on ERD, focusing on action observation (Pineda et al., 2000) and an object-oriented action (Muthukumaraswamy et al., 2004) among healthy participants. In a previous experiment, we found that MVF induced a shift in ERD toward the sensorimotor area ipsilateral to the moving hand in healthy adults, when comparing direct view feedback in the low mu, high mu, and low beta bands (Zhang and Fong, 2019). However, to the best of our knowledge, the effects of ERD on mirror illusions in stroke patients receiving mirror therapy have not been investigated before. Thus, the aim of this study was to use EEG to compare the immediate effects of a single session of MVF and evaluating ERD in the motor cortex of both hemispheres that occurred with a mirror, no mirror, and those with a covered mirror in response to unimanual arm movement in patients with chronic stroke and in their healthy counterparts. We hypothesized that ERD is associated with the subject’s observation of the mirror illusion and arm severity in patients with stroke and provides a selective index of movement-related activity that can be exclusively attributed to the discharge of mirror neurons in the motor cortex (Gallese et al., 1996).

## Materials and Methods

### Participants

The participants in this study were 11 patients with chronic stroke whom we recruited by convenience sampling from community self-help groups in Hong Kong. The study’s inclusion criteria were patients who had: (1) a neurological condition with unilateral left hemiparesis; (2) a score between levels 2 and 6 on the Functional Test of Hemiplegic Upper Extremity (FTHUE) (Fong et al., 2004), and higher scores represent a higher level of arm functioning; (3) chronic stroke, with the onset of the neurological condition having occurred more than 6 months previously; (4) the ability to understand and follow simple verbal instructions; (5) the ability to participate in a therapy session lasting at least 30 min; (6) the ability to be community ambulant, with or without aids; (7) normal or corrected-to-normal vision; and (8) right dominance before the stroke. Individuals with severe neglect and severe spasticity (Modified Ashworth Scale >3) (Bohannon and Smith, 1987) were excluded. Twenty age-matched healthy right-handed controls with normal or corrected-to-normal vision (mean age = 61.3 years; 12 males, 8 females) were also recruited by convenience sampling from social networks in the community.

The study was performed in accordance with the principles of the Declaration of Helsinki, and ethical approval was sought from the Human Subjects Ethics Committee of The Hong Kong Polytechnic University (Ref. no.: HSEARS20121012008). Only participants who had given informed written consent were included.

### Procedures

Participants performed one session of mirror therapy training for the EEG recording. The dimension of the mirror box apparatus was 16 × 17 inches, and the box was placed at the midsagittal plane of the participant. During the EEG experiment, each participant was asked to sit in a comfortable chair in front of a table, on which he/she was asked to place both arms. EEG was recorded during the three task conditions: tasks carried out in front of a mirror, tasks done with no mirror, and tasks performed in front of a covered mirror. The mirror task involved viewing a reflected image of the unaffected arm, with the affected arm at rest; the no mirror task was with the affected arm at rest while the unaffected arm was moving; and the covered-mirror task was to view a covered mirror while the affected arm was at rest and the unaffected arm was moving actively.

Each task condition consisted of two identical blocks, and each block consisted of 40 trials, with a 15-s break between the two blocks. The software E-Prime 2.0 (Psychology Software Tools, Inc., V2.0, Sharpsburg, PA, United States) was used to present the stimulus (the “ting” tone prompts). The software drove the loudspeaker, which presented one prompt every 5 s. The subject performed one “consecutive wrist flexion and extension movement” naturally, with the fullest range of motion possible, upon the onset of each prompt, until the entire task of one condition was completely finished. The brief break between each block was given to avoid possible fatigue. Between the task conditions, the investigators also allowed approximately 3 min of rest time, and they would explain to the participants the next task condition clearly during the rest times before the next task condition began.

We tested two groups: patients with stroke and age-matched healthy controls. In the control group, the participants were asked to use their dominant hands (with all being right-handed) as their active hand for movement (which was reflected into the mirror). Therefore, only stroke patients with left hemiplegia were recruited for comparison with their healthy counterparts. All participants were randomly assigned to different combinations of the three task conditions in different orders. The procedures were the same for both groups. Patients with stroke practiced the movements with the unaffected hand while watching the reflection of that unaffected hand in the mirror, whereas the controls moved their dominant hand while watching its reflection in the mirror. In the no-mirror tasks, patients with stroke were allowed to view their unaffected hand directly, and the controls viewed their dominant hand directly.

### Measurements

Electroencephalograms (EEG) were recorded with a 64-channel electrode cap, according to the International 10–20 System of Electrode Placement, connected to a SymAmps2 amplifier (Compumedics Neuroscan, Charlotte, NC, United States). During the recording, all electrodes were referenced to the left mastoid and were re-referenced to linked mastoid offline. An electrooculogram (EOG) was recorded to monitor eye blinking and movement. Both the EEG and EOG electrode impedances were kept below 5 kOhm, and the signals were sampled at 1,000 Hz. Head movement and eye fixation were well controlled.

### Data Preprocessing and Statistical Analysis

Electroencephalograms were processed offline using Scan 4.5 and CURRY 7 (Compumedics Neuroscan, Charlotte, NC, United States). Eye blink artifacts were first corrected by the regression method. Append recording was performed to append the three conditions of EEG raw data segments together as one data file. The bad channels were removed, and epochs with large muscle or otherwise strange events were also rejected. EEG data were then low-pass-filtered at 80 Hz and high-pass-filtered at 1 Hz, and downsampled to 200 Hz. Epochs from 1,000 ms before to 1,500 ms after the onset of the auditory prompts were extracted. Then eye movement artifacts were corrected using an independent component analysis algorithm (Delorme and Makeig, 2004). The artifact trials (epochs) were rejected based on higher-order statistical measures of the independent components (Delorme et al., 2001). Surface electromyography (EMG) was used to detect each wrist flexion and extension movement onset precisely.

Clean epochs were analyzed in the time–frequency domain. The event-related spectral perturbation (ERSP) method using the *newtimef* function of EEGLAB (Makeig, 1993) was used to compute the ERD power. ERSP was calculated relative to the baseline prior to each task trial. The subjects’ brain responses during each movement task were measured by the averaged ERSPs at the ipsilateral motor cortex (electrode C4 for stroke patients with left hemiplegia, i.e., ipsilesional hemisphere and ipsilateral to the moving hand) and by the contralateral motor cortex (electrodes C3 for stroke patients with left hemiplegia, i.e., contralesional hemisphere and contralateral to the moving hand but ipsilateral to the hand behind the mirror) in the participants. Then, ERSPs from 400 to 1,100 ms were averaged in the alpha-1 band (8–10 Hz), the alpha-2 band (10–12 Hz), the low beta band defined at 12–16 Hz, and the new beta band (high beta) defined as 17–35 Hz in this study for each participant and in the three task conditions. The ERSPs were computed separately in the three task conditions. An asymmetric index was calculated from the subtraction of the ERSPs between C3 and C4 (the ipsilateral motor area) to account for the difference in activity in the contralateral and ipsilateral motor areas.

A three-way repeated-measure ANOVA was performed to test the significance of the effects of groups (stroke patients vs. healthy peers) and the three task conditions (mirror vs. no mirror vs. covered mirror). *Post hoc* analysis was conducted for testing differences across time with the three task conditions. Then, a three-way ANOVA was performed with the within-subject factor for the three task conditions (real mirror vs. no mirror vs. covered mirror), hemisphere (contralateral vs. ipsilateral, here contralateral or ipsilateral to the trained hand), and the between-subjects factor for the groups (stroke patients vs. healthy peers) in the alpha-1, alpha-2, low beta, and high beta bands separately. Spearman’s correlation coefficient was used to investigate the correlation between arm severity measured by the FTHUE and ERSP values of C3 and C4 in the three task conditions in each frequency band.

## Results

After preprocessing the data for the original 11 stroke patients and 20 healthy adults, we excluded the data for seven of the healthy participants and for one stroke participant with left hemiplegia who did not present with enough clean epochs for further analysis. Ultimately, data for 10 stroke participants with left hemiplegia and 13 normal healthy right-handed counterparts were used for further analysis, and their demographics are shown in Table 1. The majority of the variables passed the Shapiro–Wilk test of normality, except that four variables violated the normality, which were in the alpha-1 and alpha-2 bands only.

**TABLE 1:** Baseline characteristics of the study population.

Variable	Stroke patients (*n* = 10)	Healthy controls (*n* = 13)	*p*
Age, mean ± SD	56.10 ± 14.35	55.54 ± 5.68	0.909^*a*^
**Gender, *n* (%)**			0.593^*b*^
Male	5 (50.00%)	7 (53.80%)	
Female	5 (50.00%)	6 (46.20%)	
Duration from stroke onset, *months* (mean ± SD)	35.80 ± 22.93	NA	NA
**Hemiplegic side, *n* (%)**			
Right	0 (0.00%)	NA	NA
Left	10 (100.0%)	NA	NA
**Recruitment site, *n* (%)**			
Hospital	0 (0.00%)	NA	NA
Self-help groups	10 (100.00%)	NA	NA
**Arm functioning, *n* (%)**			
Higher	4 (40.00%)	NA	NA
Lower	6 (60.00%)	NA	NA
FTHUE, mean ± SD	4.00 ± 1.33	NA	NA
**FMA, mean ± SD**			
UL subscore	19.60 ± 8.61	NA	NA
Hand subscore	9.30 ± 6.91	NA	NA
Total score	28.90 ± 14.91	NA	NA
**ARAT, mean ± SD**			
Grasp subscore	6.60 ± 6.36	NA	NA
Grip subscore	5.10 ± 4.73	NA	NA
Pinch subscore	5.10 ± 4.73	NA	NA
Gross subscore	4.60 ± 3.17	NA	NA
Total score	21.40 ± 19.54	NA	NA
**WMFT, mean ± SD**			
FAS subscore	27.40 ± 19.54	NA	NA
Grip subscore	6.94 ± 4.28	NA	NA

*^a^Independent t-test; ^b^Chi-square test; ARAT, Action Research Arm Test; FMA, Fugl-Meyer Assessment; UL, Upper Extremity Score; FTHUE-HK, Functional Test for Hemiplegic Upper Extremity-Hong Kong version; WMFT, Wolf Motor Function Test; FAS, Functional ability score.*

**FIGURE 1:**
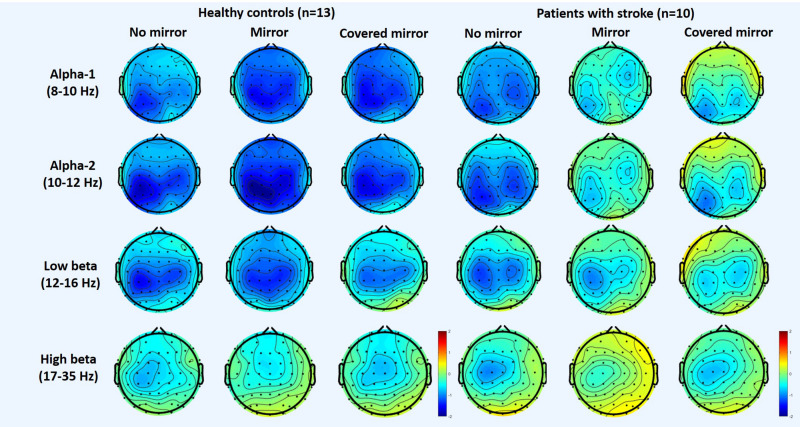
Topography of the alpha and beta rhythms in three unimanual hand movement task conditions: with mirror, with no mirror, and with covered mirror, in two groups: stroke patients and healthy controls.

Figure 1 shows the topography of the alpha and beta band rhythms in the unimanual hand movement task during the three task conditions (with a mirror vs. with no mirror vs. with a covered mirror) in the group of stroke patients and in the age-matched controls. Table 2 summarizes the means and standard deviations of the asymmetric index and ERSP power in alpha-1, alpha-2, low beta, and high beta in the three task conditions.

**TABLE 2:** The means and standard deviations of asymmetric index and ERSP power in each of the three task conditions in patients with stroke and health controls.

Population	Stroke patients			Healthy controls		
Condition	No mirror	Mirror	Covered mirror	No mirror	Mirror	Covered mirror
***Alpha-1***						
C3	−1.08 (1.13)	−0.61 (1.05)	−0.41 (1.20)	−1.07 (1.61)	−1.36 (1.23)	−1.48 (1.35)
C4	−1.15 (1.39)	−0.62 (1.01)	−0.33 (1.20)	−0.97 (1.60)	−1.35 (1.32)	−1.18 (1.54)
Asymmetric index	0.07 (0.64)	0.01 (0.42)	−0.09 (0.94)	−0.11 (0.59)	−0.01 (0.52)	−0.30 (0.64)
***Alpha-2***						
C3	−1.29 (0.85)	−0.68 (1.27)	−0.54 (1.00)	−1.38 (1.42)	−1.56 (1.31)	−1.53 (1.11)
C4	−1.21 (1.12)	−0.58 (1.18)	−0.54 (1.03)	−1.34 (1.30)	−1.55 (1.40)	−1.30 (1.34)
Asymmetric index	−0.08 (0.55)	−0.10 (0.54)	0.00 (0.49)	−0.05 (0.61)	−0.01 (0.54)	−0.24 (0.59)
***Low beta***						
C3	−1.29 (1.01)	−0.96 (1.25)	−0.58 (0.82)	−1.33 (0.93)	−1.24 (1.10)	−1.06 (0.90)
C4	−1.02 (1.22)	−0.56 (1.20)	−0.67 (0.90)	−1.24 (0.76)	−1.24 (1.05)	−1.07 (1.00)
Asymmetric index	−0.27 (0.66)	−0.40 (0.66)	0.09 (0.67)	−0.09 (0.51)	0.00 (0.40)	0.01 (0.58)
***High beta***						
C3	−0.93 (0.99)	−0.33 (0.87)	−0.74 (0.87)	−0.76 (0.90)	−0.41 (0.65)	−0.62 (0.99)
C4	−0.18 (1.16)	0.10 (0.91)	−0.24 (0.76)	−0.51 (0.75)	−0.36 (0.82)	−0.45 (1.04)
Asymmetric index	−0.76 (0.85)	−0.43 (0.29)	−0.50 (0.28)	−0.25 (0.40)	−0.05 (0.38)	−0.17 (0.32)

**FIGURE 2:**
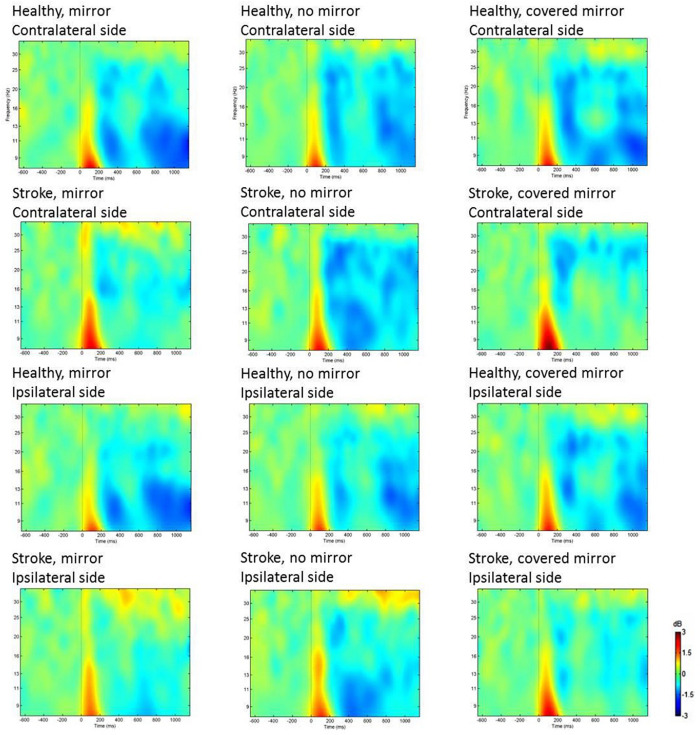
Comparison of ERSPs at electrodes C3 (contralateral motor area) and C4 (ipsilateral motor area) in three unimanual hand movement task conditions: with mirror, with no mirror, and with covered mirror, in two groups: stroke patients and healthy controls, with ERD presented in color blue.

In the alpha-1 and alpha-2 bands, there was neither a significant main effect nor an interaction effect of *tasks* × *group* × *hemisphere* (Figure 2). The results of ANOVA revealed a significant effect from task in the low beta band (12–16 Hz) [*F*(1,21) = 3.775, *p* = 0.031]. Moreover, in the high beta band (17–35 Hz), the three-way ANOVA showed a significant interaction effect from *hemisphere* × *group* [*F*(1,21) = 7.698, *p* = 0.011], a significant main effect from hemisphere [*F*(1,21) = 24.299, *p* < 0.0001], and a significant main effect from the task [*F*(2,42) = 3.216, *p* = 0.050]. There was a marginal interaction effect from *task* × *hemisphere* in the high beta band, with an *F* ratio of *F*(2,42) = 3.187, *p* = 0.051.

Further examination of the effect of *task* × *hemisphere* showed that in the contralateral hemisphere, C3 (contralateral motor area) had more suppression in the no-mirror and covered-mirror conditions than they did in the mirror condition in the high beta band (17–35 Hz), with an F ratio of *F*(2,42) = 6.003, *p* = 0.005.

An ANOVA was carried out to explore the effects of the task condition and the group on the asymmetry index in the alpha-1, alpha-2, low beta, and high beta bands. Significant main effects of the group on the asymmetry index were found in the high beta band [*F*(1,21) = 7.698, *p* = 0.011], with the stroke patients showing greater asymmetry than the healthy controls did (Figure 2).

**FIGURE 3:**
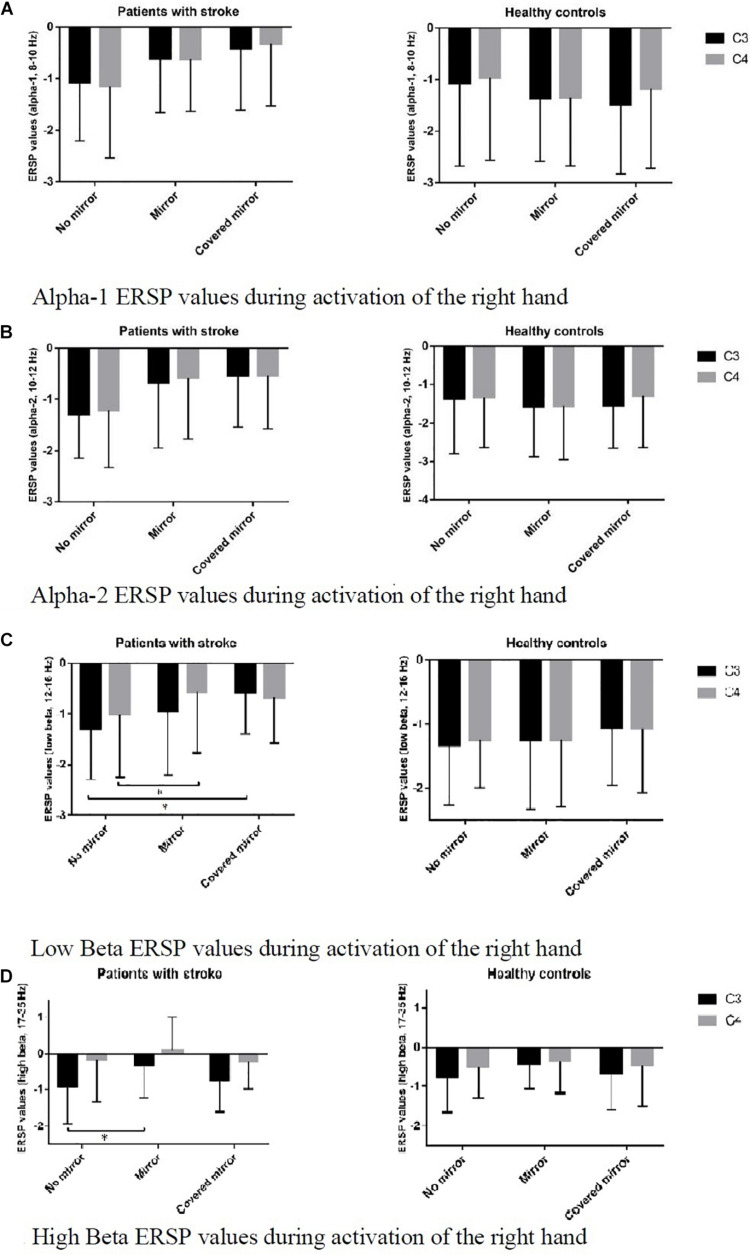
ERSP values of C3 and C4 in three task conditions in each frequency band, in two groups: stroke patients and healthy controls. **(A)** Alpha-1 ERSP values during activation of the right hand. **(B)** Alpha-2 ERSP values during activation of the right hand. **(C)** Low Beta ERSP values during activation of the right hand. *p < 0.05. **(D)** High Beta ERSP values during activation of the right hand. *p < 0.05.

Figure 3 shows the ERSP values of C3 and C4 in 3 task conditions in each frequency band in the two groups. *Post hoc* pairwise comparison was performed in the two bands – C3 and C4, and the asymmetric index (i.e., C3–C4). Regarding the high beta band in patients with stroke, we found a significant difference in C3 ERD between the no-mirror and mirror conditions, i.e., more suppression under the no-mirror condition in C3 compared to that of the mirror condition (*p* = 0.022) (Figure 3D). Regarding the low beta band in patients with stroke, we found a significant difference in C3 ERD between the no-mirror and covered-mirror conditions, i.e., more suppression in C3 under the no-mirror condition compared to that of the covered-mirror condition (*p* = 0.014), as well as a significant difference in C4 ERD between the no-mirror and mirror conditions, i.e., more suppression in C4 under the no-mirror condition compared to that under the mirror condition (*p* = 0.032) (Figure 3C). However, only a significant difference in the asymmetric index was found between the no-mirror and mirror conditions in healthy controls, i.e., more asymmetric under the no-mirror condition compared to the mirror condition (*p* = 0.039).

Table 3 shows the correlation between the levels of arm severity with ERSP power in the three task conditions in patients with stroke. Regarding the low beta band, there was a significant moderate correlation between the levels of arm severity measured by the FTHUE with the ERSP power in the no-mirror condition in C4 (*r* = −0.731) and the asymmetric index (*r* = 0.675). The levels of arm severity were found to correlate strongly with ERSP power in the mirror condition in C4 (*r* = −0.700). In contrast, the levels of arm severity correlated strongly with ERSP power in C3 in the no-mirror (*r* = −0.706) and mirror (*r* = −0.737) conditions as well as in C4 in the mirror (*r* = −0.712) and covered-mirror (*r* = −0.700) conditions.

**TABLE 3:** Correlation between levels of arm severity with ERSP power in three task conditions in patients with stroke.

Condition	No mirror			Mirror			Covered mirror		
Position	C3	C4	AI	C3	C4	AI	C3	C4	AI
***Alpha-1***									
FTHUE	−0.056	−0.099	0.353	−0.074	−0.359	0.489	0.074	0.068	0.006
***Alpha-2***									
FTHUE	−0.248	−0.050	0.198	−0.384	−0.570	0.279	0.161	−0.223	0.440
***Low beta***									
FTHUE	−0.396	−0.731*	0.675*	−0.601	−0.700*	0.365	−0.155	−0.539	0.458
***High beta***									
FTHUE	−0.706*	−0.570	−0.204	−0.737*	−0.712*	0.229	−0.576	−0.700*	0.012

*p < 0.05; FTHUE, levels of arm severity measured by the Functional Test of the Hemiplegic Upper Extremity; AI, asymmetric index.

## Discussion

This study used EEGs to investigate the immediate effects of a single session of MVF in the motor cortex compared with the effects occurring without a mirror during unimanual arm movements in patients with chronic stroke and in their healthy peers. EEG showed similar ERD to MVF in the alpha bands in patients with stroke compared to those found in healthy controls, although the ERSP power in patients with stroke was much lower, and that the attenuation of the low beta ERD was greater over the contralateral hemisphere than it was over the ipsilateral hemisphere in both no-mirror and mirror conditions in patients with stroke.

A reduction of the hemispheric asymmetry was found in patients with stroke in the high beta band during unimanual movement of the non-affected arm. In this study, the magnitude of MVF-induced low beta attenuation in contralesional hemisphere is similar to the finding in patients with right hemispheric stroke (Bartur et al., 2018) except that we also found more suppression in C4 over the ipsilesional hemisphere under the no-mirror condition compared to that under MVF. This finding was consistent with our review that MVF resulted in a shifted activation toward the ipsilesional hemispheres in patients who have had strokes; hence, a more symmetrical state between the two hemispheres may be achieved (Zhang et al., 2018). Stroke is a chronic and disabling disease that is common among the adult population. Recent studies have found that brain recovery can still take place long after the original event, if the patient is undergoing active rehabilitation. Mirror therapy emerged 10 years ago as an intervention for upper limb hemiparesis following stroke and has the advantage of being very inexpensive as well as simpler and less labor-intensive than other types of intervention. In stroke patients, one possible explanation for paralysis being unlearned after watching the mirror illusion during movement is that a residue of mirror neurons has survived the stroke, but it is either dormant or its activity is inhibited and does not reach the threshold. Another possibility is that the motor areas may have become temporarily inactive because the patient’s visual feedback loop has closed, and the mirror image might provide sufficient visual input to revive those motor neurons (Ertelt et al., 2007).

We know that M1 excitability is modulated simultaneously both by hemiplegic limb movement and by observation of the movement of the non-affected limb as reflected in the mirror during MVF, with the ipsilateral M1 becoming more active (Garry et al., 2005), as MVF decreases the motor threshold through a reduction in interhemispheric inhibition (Carson and Ruddy, 2012) and a reduction of intracortical inhibition (Lappchen et al., 2012). A key mechanism hypothesized to explain these beneficial effects is that the mirror illusion transiently decreases the asymmetric activities of movement-related desynchronization in the beta frequency range (Rossiter et al., 2015).

In this study, it was interesting to find that there was a significant main effect of groups in both the contralateral and ipsilateral motor areas, and particularly in the contralateral motor area C3, in the age-matched controls and stroke participants’ groups in the high beta band. Traditionally, we have believed in bilateral mu suppression during movement execution (Frenkel-Toledo et al., 2013). Previous studies also showed that MVF operates in a different manner – in different frequency bands and involving either or both hemispheres (Bartur et al., 2018).

We also found overall reduced ERD in both hemispheres in patients with stroke in various frequency bands compared to that of their healthy peers. This is consistent with other studies that the ERD induced by observation is very limited in stroke patients, in both ipsilesional and contralesional hemispheres. It is unclear why ERD does not occur in stroke patients partly because of the lower functional recovery of the affected arm. Our correlation analysis revealed that the magnitude of ERSP power correlated significantly with arm severity in the low and high beta bands in patients with stroke. The minus sign indicated that it was a negative relationship. Results revealed that higher levels of the FTHUE and higher functioning of the upper extremity (or less severe arm impairment) were associated with lower values of the ERSP power in the no-mirror and mirror conditions at the ipsilesional C4, i.e., stronger suppression at the low beta. The positive relationship of the levels of arm severity with the asymmetric index (a lower value showing more symmetry between the hemispheres and a shifted activation toward the ipsilesional hemisphere) in the low beta in the no-mirror condition revealed a higher asymmetry between the hemispheres, i.e., the ERSP values at the contralesional C3 was higher than that at the ipsilesional C4, in patients with higher arm functioning. At the high beta, similar findings were noted in the mirror and covered-mirror conditions in C4, but a significant negative correlation was also found in the contralesional C3 in the no-mirror and mirror conditions, which reflects likely the motor execution of the moving hand. Even though the underlying neural network cannot be fully understood at this stage, the reduced ERD in both hemispheres in patients who have had strokes might cause problems in action observation and movement preparation for execution in their motor learning, hence affecting their motor recovery. Furthermore, when patients suffer damage to their inferior parietal lobe or inferior frontal gyrus, the MNS as indicated by the ERD induced over their contralesional sensorimotor cortex through observation is attenuated too (Frenkel-Toledo et al., 2014, 2016; Perry et al., 2017). As a single MVF session was found to reduce the magnitude of ERD bilaterally in stroke patients in the current study, further investigation of the neurophysiological effects of MVF application in randomized controlled studies is warranted.

The results of this study show that there was an MVF-induced ERD attenuation in the high and low beta bands, and that the rhythm suppression was greater over the contralateral hemisphere (i.e., contralesional in stroke) than it was over the ipsilateral hemisphere in the low beta in stroke participants. This finding is consistent with the results of another study using magnetoencephalography (MEG) that the ERD in M1 beta decreased more in the bilateral mirror condition than in the bilateral no-mirror and unimanual mirror conditions (Tai et al., 2020), and that the ERD areas in both the alpha and beta were larger under the reciprocal mirror condition, followed by bilateral and no-mirror conditions (Chang et al., 2019). In a recent MEG study (Rossiter et al., 2015), similar results of movement-related beta desynchronization were found in patients and their healthy controls, but those observations differed from the results of another study (Frenkel-Toledo et al., 2013) that found in an EEG analysis a more pronounced suppression over the right hemisphere than over the left hemisphere sites during observation of action, regardless of the hand that moved (right or left hand). Our previous study demonstrated that a shift in sensorimotor asymmetry toward the contralateral hemisphere was induced by MVF immediately, in alpha-1, alpha-2, and beta-1 bands in healthy right-handed adults (Zhang and Fong, 2019). Another study showed that the hemispheric symmetry was reduced during non-affected arm movement in the mirror condition after a single session of MVF in patients with subacute stroke (Bartur et al., 2018). These indicated that MVF might contribute to stroke recovery by revising the interhemispheric imbalance caused by stroke due to the activation of the MNS, hence promoting motor relearning in stroke patients (Zhang et al., 2018). Our findings concur with a previous study that the low beta ERD in both hemispheres has the potential to be used as a neurophysiological marker for the MVF treatment outcome for improving stroke patients’ recovery in future studies (Bartur et al., 2018).

### Limitations of the Study

This was a cross-sectional study, the change of the amount of MVF-induced ERD attenuation across time had not been investigated, and that its relationships with motor severity and functional prognosis had not been revealed. The results of this study were based on a small sample from the chronic stroke population, so a bias was possible in the subject recruitment, which was based on convenience sampling. Since the participants with stroke were recruited through self-help organizations in the community, we did not know the detailed lesion site from the participants. Our results were based on a group of stroke patients with right hemispheric damage and had not compared their performance with that of patients with left hemispheric brain damage. This study only considered the ERD phenomena on the primary motor areas; other brain regions such as the parietal areas in the MNS had not been investigated. The hemispheric asymmetry in the low beta under MVF was particularly more salient following right hemispheric damage (Bartur et al., 2018). Although the MVF leading to an instant ERD might have been associated with facilitation of M1 (ipsilateral to the moving hand that was mirrored), our findings must be interpreted with care, and we caution that the variance in the movement task conditions, which was unimanual and without involvement of the affected hand, as well as the levels of stroke impairment, may have complicated the overall interpretation of the results. Moreover, the demographics of the older adult control participants did not closely match with those in the stroke group.

## Conclusion

Our study shows that the laterality of bilateral sensorimotor ERD during unimanual arm movement could be mediated by the recognition of MVF in both stroke patients with right hemispheric damage and healthy controls. The findings of this study shed light for neurophysiological investigations during motor training in stroke rehabilitation. Further study should investigate the effects of training, both by MVF and bimanual arm training, with the goals of reestablishing the hemispheric balance that has been disrupted by an insult in the motor cortex and consequently of improving patient readiness in lateralized potential for motor recovery. The use of ERD in the low beta band in the motor cortex as a neurophysiological marker to indicate the relationships between the amount of MVF-induced ERD attenuation and motor severity and functional prognosis in patients with stroke, and an outcome indicator for improving patients’ neuroplasticity in clinical trials is warranted to be explored in the future.

## Data Availability Statement

The raw data supporting the conclusions of this article will be made available by the authors, without undue reservation.

## Ethics Statement

The studies involving human participants were reviewed and approved by the Human Subjects Ethics Committee of The Hong Kong Polytechnic University (Ref. No.: HSEARS20121012008). The patients/participants provided their written informed consent to participate in this study.

## Author Contributions

KNKF and KHT were involved in the conception and design of the study and conducted the experiment. KNKF and CSFY collected the data. KNKF and JJQZ wrote up and edited the manuscript. All authors approved the submission of the final version of the manuscript.

## Conflict of Interest

The authors declare that the research was conducted in the absence of any commercial or financial relationships that could be construed as a potential conflict of interest.
